# Visualisation and analysis of hepatitis C virus non-structural proteins using super-resolution microscopy

**DOI:** 10.1038/s41598-018-31861-0

**Published:** 2018-09-11

**Authors:** Christopher Bartlett, Alistair Curd, Michelle Peckham, Mark Harris

**Affiliations:** School of Molecular and Cellular Biology, Faculty of Biological Sciences and Astbury Centre for Structural Molecular Biology, University of Leeds, Leeds, LS2 9JT UK

## Abstract

Hepatitis C virus (HCV) RNA replication occurs in the cytosol of infected cells within a specialised membranous compartment. How the viral non-structural (NS) proteins are associated and organised within these structures remains poorly defined. We employed a super-resolution microscopy approach to visualise NS3 and NS5A in HCV infected cells. Using single molecule localisation microscopy, both NS proteins were resolved as clusters of localisations smaller than the diffraction-limited volume observed by wide-field. Analysis of the protein clusters identified a significant difference in size between the NS proteins. We also observed a reduction in NS5A cluster size following inhibition of RNA replication using daclatasvir, a phenotype which was maintained in the presence of the Y93H resistance associated substitution and not observed for NS3 clusters. These results provide insight into the NS protein organisation within hepatitis C virus RNA replication complexes and the mode of action of NS5A inhibitors.

## Introduction

Hepatitis C virus (HCV) is a major human pathogen estimated to infect ~170 million people worldwide and is a leading cause of liver disease^1^. Direct-acting antiviral (DAA) treatments have improved clinical outcomes for infected patients, however some virus strains are less responsive and no vaccine is available^2^. HCV is an enveloped virus with a positive-sense RNA genome, and a member of the *Hepacivirus* genus within the *Flaviviridae* family^3^. Translation of the HCV genome produces a single polyprotein, which is subsequently cleaved by cellular and viral proteases into three structural (core, E1, E2) and seven non-structural (NS) proteins (p7, NS2, NS3, NS4A, NS4B, NS5A and NS5B)^4^.

HCV RNA replication is coordinated by NS3–5B, which is both necessary and sufficient^5^, within a convoluted “membranous web” (MW) in the cytoplasm of infected cells^6^. NS3/4A is a protease/helicase required for HCV polyprotein cleavage and RNA secondary structure unwinding^7,8^. NS4B is involved in the formation of the MW^6^ along with NS5A, a multi-functional phosphoprotein^9^. NS5B is the RNA-dependent RNA polymerase^10^. The MW is constructed from single, double and multi-membrane vesicles which are derived from the endoplasmic reticulum (ER)^11–13^. Remodelling of intracellular membranes is characteristic of positive-sense RNA viruses and provides specialised “replication factories” for viral RNA replication and virion assembly (reviewed in^14^). Double membrane vesicles (DMVs) are the predominant species observed during HCV infection and their production correlates with RNA replication kinetics^12^. HCV DMVs are typically 100–300 nm in diameter and are distributed throughout the cytoplasm of infected cells^12,15^, as demonstrated by electron microscopy (EM). This is consistent with the subcellular distribution of diffraction-limited puncta observed by fluorescence microscopy for both NS5A and NS3^16^. Further, immunogold labelling confirmed an association between NS3, NS5A and dsRNA with DMVs^12,17^, which exhibit replicase activity when purified^11^.

NS5A is an essential component involved in HCV replication with a number of known interaction partners, such as HCV RNA^18^, other NS proteins^19^, cellular proteins^20^ and cytosolic lipid droplets, cellular lipid storage organelles required for HCV virion assembly^21^. The DMVs within the MW are the proposed site for HCV RNA replication. However, there is little evidence describing precisely how the NS proteins are organised with these structures, and therefore where the site of HCV RNA replication occurs. Daclatasvir (DCV) is a small molecule inhibitor of NS5A, effective at pM concentrations^22^. It is known that two mutations in domain I of NS5A, L31V and Y93H, confer resistance to DCV, indicating a likely DCV interaction site^22^. However, it is unclear how DCV inhibits NS5A, as NS5A has no known enzymatic function. As a symmetric compound, DCV has been suggested to preferentially bind dimers of NS5A, possibly stabilising them^23,24^. Alternatively, DCV may interfere with NS5A binding to RNA^25^ or its association with membranes^26^. Fluorescence microscopy revealed that NS5A puncta redistribute to the perinuclear region of cells during 8 h DCV treatments^27,28^. Longer treatments of up to 24 h identified that NS5A relocated to lipid droplets, and the MW collapsed as the number of DMVs reduced^29^.

To better understand, and determine the organisation of NS5A and NS3 proteins within clusters in HCV infected cells we have used the single molecule localisation microscopy (SMLM) approach of direct stochastic optical reconstruction microscopy (dSTORM)^30,31^. dSTORM, a type of ‘super-resolution microscopy’ allows the localisation of fluorescently labelled molecules to be identified with precisions below 20 nm laterally from fluorescence labelling^30–32^ and ~50 nm axially by manipulating the shape of the point spread function^33–36^. This is a large improvement over standard widefield fluorescence microscopy which can only achieve resolutions of ~200 nm laterally and ~500 nm axially at best^37^. dSTORM and the related approach of photoactivated light microscopy (PALM) have provided insight into the organisation of proteins in complex assemblies such as adhesion complexes^38^, the nuclear pore^39^, mammalian primary cilia^40^ the cytoskeletal organisation in axons^41^, and the organisation of HCV proteins around the viral assembly site^42^. Here, we were able to use 3D dSTORM to analyse the sizes of clusters of NS5A and NS3, resolving differences of 10–30 nm in the size of sub-100 nm clusters. We developed the use of a clustering-based image segmentation algorithm, density-based spatial clustering of applications with noise (DBSCAN)^43^, which allowed us to characterise the size and morphology of NS5A and NS3 protein clusters. Finally, we investigated whether DCV treatment had any effect on the size of NS5A protein clusters, which might be expected if this drug affects NS5A dimerisation or membrane association. From these investigations we identified a difference in protein cluster size between two NS proteins involved in HCV RNA replication, and a significant reduction of NS5A protein cluster size following DCV treatment.

## Results

### 3D-dSTORM imaging of hepatitis C virus infected cells reveals that NS5A and NS3 organise into clusters

**Figure 1:**
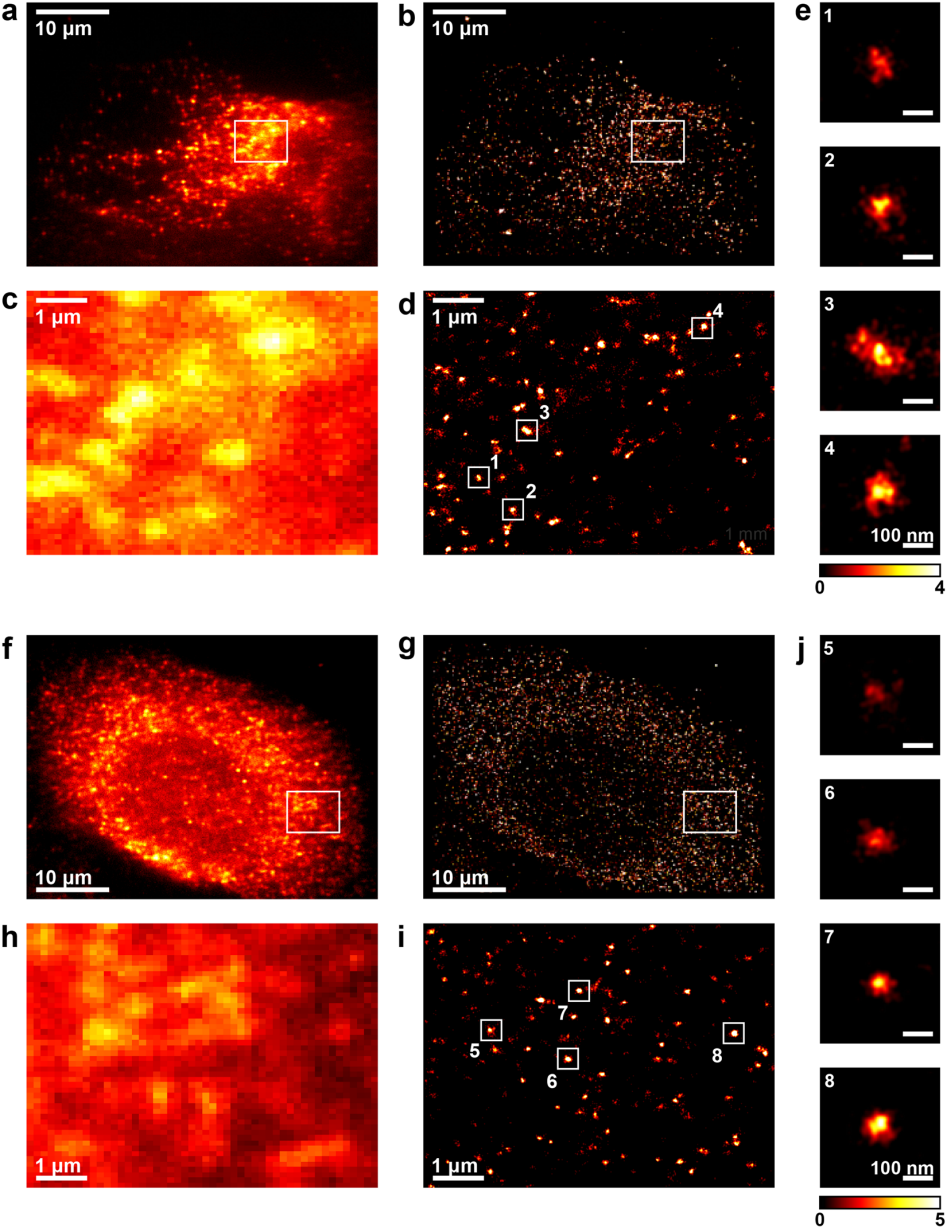
NS5A and NS3 fluorescent foci are resolved as clusters of localisations by 3D-dSTORM. (**a**–**e**) NS5A, (**f**–**j**) NS3. (**a**,**f**) Wide-field fluorescence images of Huh7 cell 24 hours post-infection with JFH-1 and immunostained for NS5A or NS3. (**b**,**g**) 3D-dSTORM images of cell in (**a**,**f**). (**c**,**h**) Projections of 2 μm-thick volumes, 100 nm histogram bins. Regions of interest within (**a** and **f)** at higher magnification. (**d**,**i**) 3D-dSTORM images of regions shown in (**c**,**h)**. Projections of 2 μm-thick volumes, 10 nm histogram bins. (**e**,**j**) Individual clusters of localisations (numbered in **d**,**i**). Projections of 1 μm-thick volumes, 5 nm histogram bins. Colour bar indicates localisation density per histogram bin. 3D-dSTORM images are sum projections over depth (*z*) smoothened with a Gaussian filter, σ = 20 nm.

Wide-field fluorescence imaging of NS5A in Huh7 cells (a hepatocarcinoma cell line), infected with the JFH-1 strain of hepatitis C virus for 24 h, showed that this protein localised to diffraction-limited foci, distributed throughout the cytoplasm (Fig. 1a). Using 3D-dSTORM, these diffraction-limited foci of NS5A were resolved as numerous spatially distinct clusters of localisations (Fig. 1b–d), with sizes varying from around 100 to 300 nm (Fig. 1e). These clusters are within the size range of DMVs observed by electron microscopy^12^, were not observed in naïve Huh7 cells (see Supplementary Fig. S1), and are likely to represent sites of HCV replication complexes.

Similarly to NS5A, wide-field fluorescence imaging demonstrated that NS3 localised to diffraction-limited foci that were distributed throughout the infected cell cytoplasm (Fig. 1f), in Huh7 cells infected with JFH-1 for 24 hours. Using 3D-dSTORM imaging of the same cells (Fig. 1g), NS3 localisations were again resolved as discrete clusters (Fig. 1h,i), and these exhibited a similar size range and morphology (Fig. 1j) to NS5A clusters (Fig. 1e).

### Developing DBSCAN to analyse cluster sizes for NS5A and NS3

**Figure 2:**
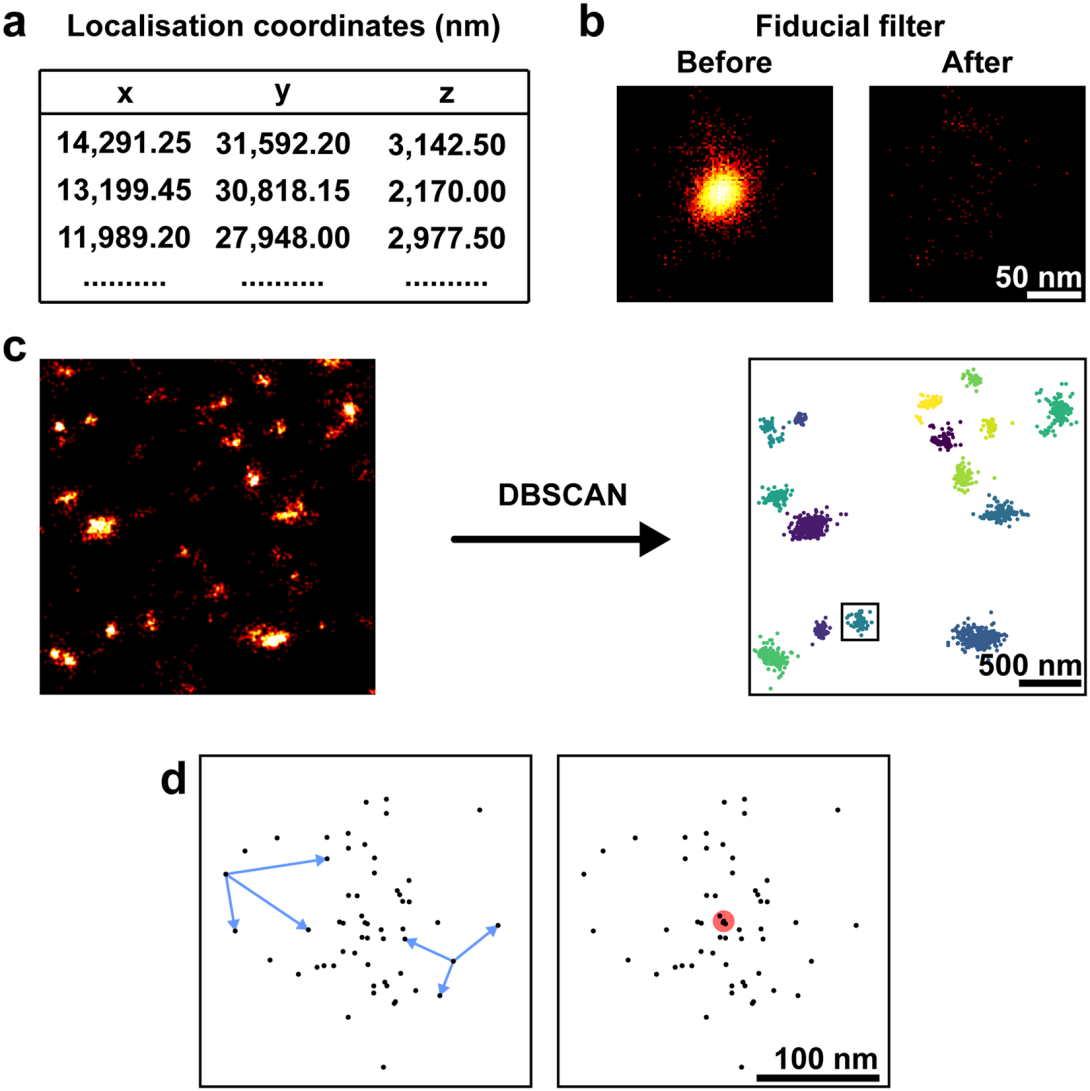
Image analysis pipeline. (**a**) 3D-dSTORM *x*-*y*-*z* localisation coordinates extracted from *palm3d* output files. (**b**) Fiducial marker localisations removed from the coordinate list. Region containing localisations of a fiducial marker shown with logarithmic look-up table. (**c**) Localisations classified into clusters by DBSCAN. Localisations not found in a cluster were removed from the analysis. Localisations before DBSCAN analysis shown with linear look-up table. Localisations clustered by DBSCAN coloured by their cluster identity. (**d**) A single cluster (box in **c**). Intra-cluster distances (blue arrows) and the centroid (red circle) for each cluster were used as described.

To segment and characterise the clustered localisations for NS5A and NS3 obtained in the 3D-dSTORM images, we applied DBSCAN^43^, implemented in Python (Fig. 2). Localisation coordinates were first extracted from the output of *palm3d* software^34^ (Fig. 2a). Any fiducial markers (used for drift tracking, see Methods) in a region of interest were filtered (see Methods), to avoid their misidentification as a protein cluster, and the resulting filtered image was inspected (Fig. 2b). Localisations within the resulting dataset were considered clustered by DBSCAN if they contained at least 30 neighbours within a 150 nm search radius (Fig. 2c). These parameters were chosen to reduce the detection of self-clustering around a single fluorophore undergoing repeated localisation in dSTORM^44^, but not so big that clusters merged together. Measurements of each cluster (Fig. 2d) were used for quantitative analysis. All clustering and subsequent analysis used 70.6 µm3 cytosolic regions of interest from three independent cells.

As in previous work^45^, we registered clusters by their centroid position and averaged the clusters over all cells to increase the signal-to-noise ratio for the localisation distribution. The cluster diameter for individual protein clusters was measured by calculating the median pairwise Euclidean distance in *x*-*y* between all localisations, disregarding the *z*-coordinate, which has lower localisation precision (see Supplementary Table 1). Cluster diameter is therefore a single measurement which does not rely upon a parametric fit of the localisation distribution and is relatively insensitive to cluster shape. For comparison, a spherical Gaussian distribution of localisations has cluster diameter = 0.71 × FWHM (full-width half-maximum) (see Supplementary Fig. S3).

An observed cluster is a convolution of the actual distribution of labelled proteins with the localisation precision for the fluorescent dye molecules and the effect of linkage error from antibody labelling^46^. Therefore, an upper limit on localisation precision for the fluorescent labels could be determined by measuring the spread of localisations within the smallest clusters (see Supplementary Fig. S4). A Gaussian fit to the line profiles of the smallest NS3 clusters revealed a localisation precision smaller than 20 nm for *x*-*y*, and 93 nm for *z*.

### Quantitative clustering analysis reveals NS5A clusters are larger than NS3 clusters

**Figure 3:**
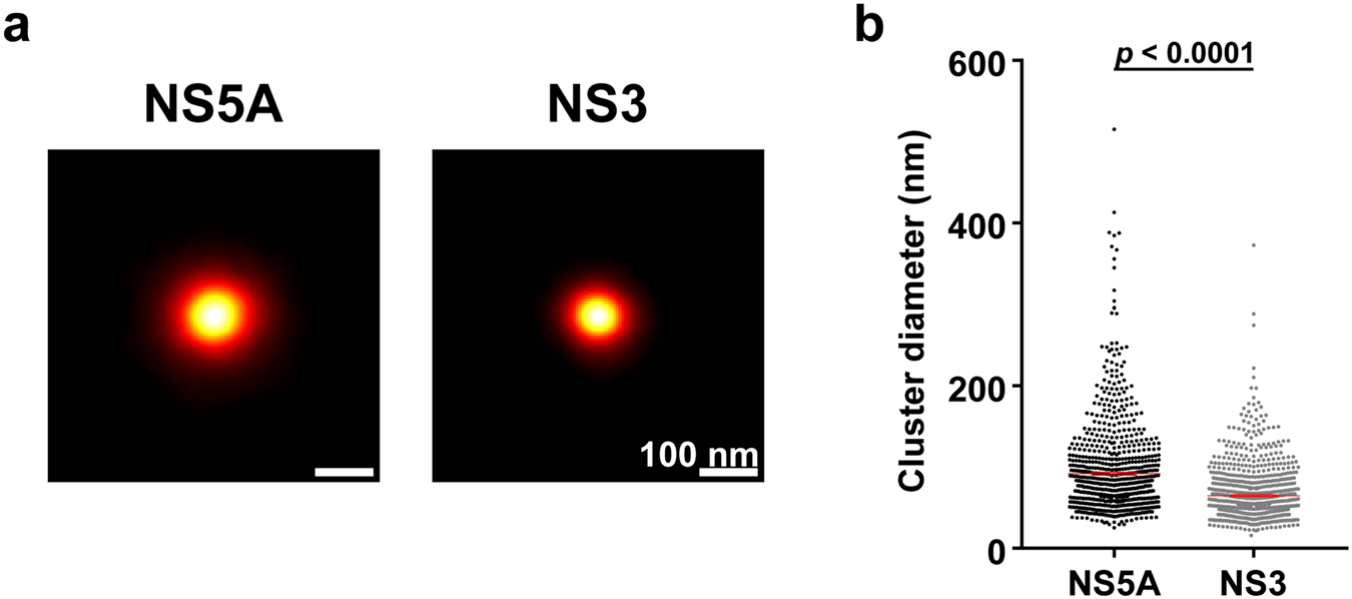
NS5A and NS3 clusters exhibit different sizes. (**a**) Average clusters of NS5A and NS3 from JFH-1 infected cells. (**b**) NS5A and NS3 cluster diameters. Mean and standard error of the mean are shown. Statistical significance determined by two-tailed Student’s *t*-test with Welch’s correction.

Averaging all clusters for both proteins revealed that NS5A clusters were larger than NS3 clusters on average (Fig. 3). The range of cluster diameters was similar for NS5A and NS3 clusters (Fig. 3b). However, the mean cluster diameter for NS3 (64.7 ± 1.1 nm; mean ± SEM; *n* = 943) was significantly smaller than that for NS5A (91.8 ± 1.9 nm; *n* = 891) (*t* = 12.6, *p* < 0.0001, Student’s *t*-test with Welch’s correction). Although both NS proteins are involved in HCV RNA replication, 3D-dSTORM revealed a difference in their organisation within HCV infected cells.

We additionally considered the possibility that the difference between NS5A and NS3 cluster size arose from differences between their labelled antibodies. Using an alternative NS5A antibody (Virostat - see methods), similar clusters of localisations were identified in infected cells (see Supplementary Fig. S5). The average NS5A cluster size using the alternative antibody was measured at 70.7 ± 2.3 nm (mean ± SEM; *n* = 405). This is in disagreement with our initial observations for NS5A and is closer in size to our observations with NS3. Therefore, we cannot eliminate the possibility of antibody specific difference in our observations between NS5A and NS3. However, under the same experimental conditions fewer clusters were resolved using the Virostat antibody, indicating that 3D-dSTORM can resolve differences between antibodies against the same target.

Considering the possibility of different antibodies identifying functionally distinct NS5A cluster species, experiments were conducted to label and image HCV RNA. Both metabolic labelling using 5-ethynyl uridine (data not shown) or RNA FISH^47^ (data not shown) were unsuccessful. Use of dsRNA antibody^12^ labelling did reveal reactivity but this did not colocalise with NS5A (see Supplementary Fig. S6) suggesting that it is not in fact a good marker for replication complexes. Further investigations are therefore required to understand these differences between phenotypes and confirm the cluster structures as replication complexes. However, our observations are consistent with the size, distribution and shape of membrane vesicles observed by others^12^.

### Cells stably harbouring sub-genomic replicons form larger NS5A protein clusters

**Figure 4:**
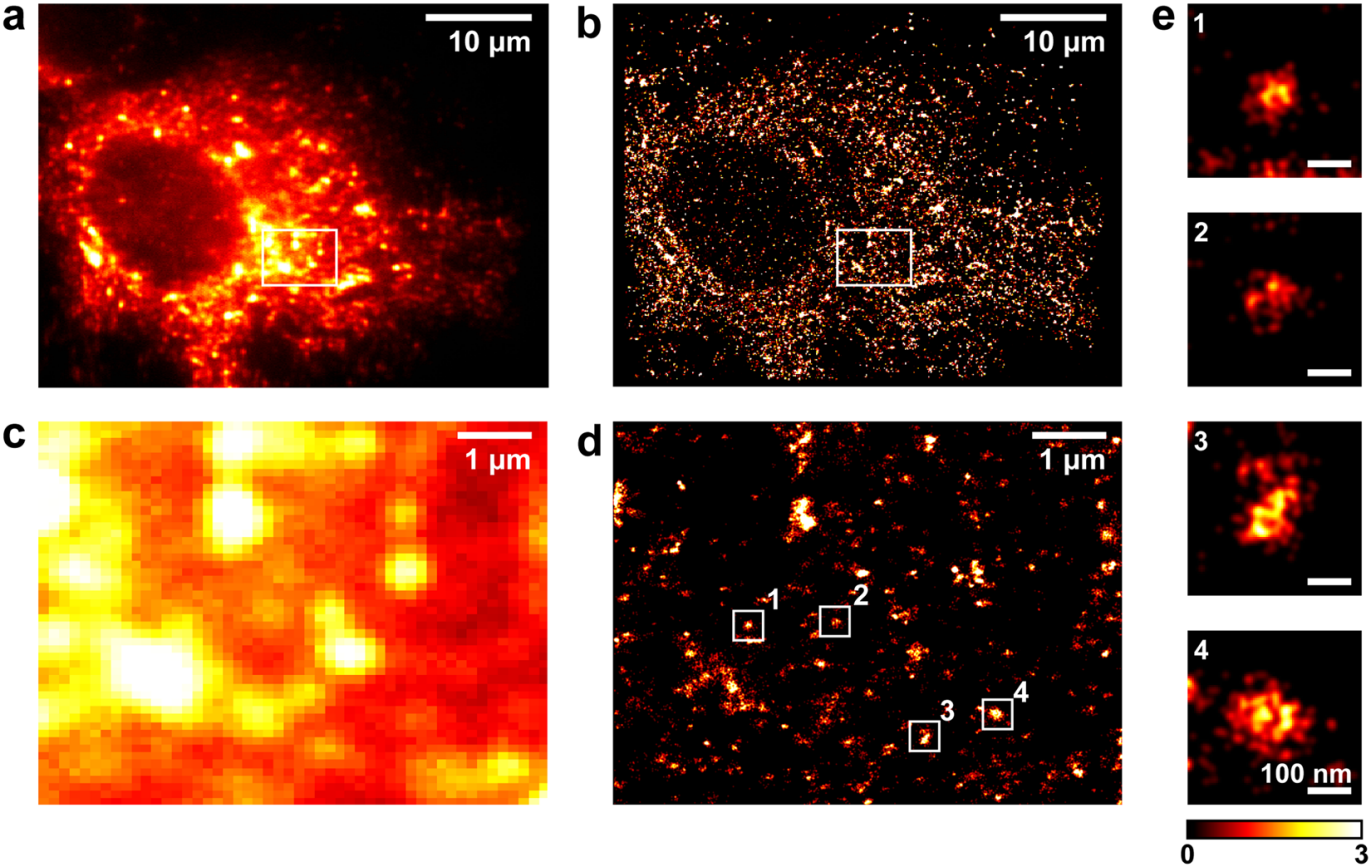
NS5A clusters from SGR-JFH-1 stable cell lines are larger than infected cells. (**a**) Wide-field fluorescence image of Huh7 cells stably harbouring SGR-Neo-JFH-1 and immunostained for NS5A. (**b**) 3D-dSTORM image of cell in (**a)**. Projection of 2 µm-thick volume, 100 nm histogram bins. (**c**) Region of interest within (**a)** at higher magnification. (**d**) 3D-dSTORM image of region shown in (**c**). Projection of a 2 µm-thick volume, 20 nm histogram bins. (**e**) Individual clusters of localisations (numbered in **d**). Projections of 1 µm-thick volumes, 5 nm histogram bins. Colour bar indicates localisation density per histogram bin. 3D-dSTORM images are sum projections over depth (*z*) smoothened with a Gaussian filter σ = 20 nm.

To analyse NS5A protein clusters in the absence of the core-dependent localisation of NS5A to lipid droplets, required for virus assembly and release^21^, 3D-dSTORM imaging was conducted on cells stably harbouring a sub-genomic replicon (SGR) (Fig. 4).These constructs contain the HCV NS proteins but not the structural proteins necessary for HCV particle assembly and release^5^. Wide-field and 3D-dSTORM imaging revealed a distribution of NS5A throughout the cytoplasm (Fig. 4a,b), consistent with our findings from HCV infected cells (Fig. 1). Areas of fluorescence were resolved as numerous discrete clusters of NS5A localisations (Fig. 4c,d), and individual NS5A clusters revealed a similar morphology to those observed during HCV infection (Fig. 4e). The mean NS5A cluster size for cells stably harbouring SGR’s was measured as 108.5 ± 1.8 nm (mean ± SEM; *n* = 1724), significantly larger than observed for cells infected with HCV for 24 h.

### Treatment of HCV infected cells with DCV decreases the size of NS5A protein clusters

**Figure 5:**
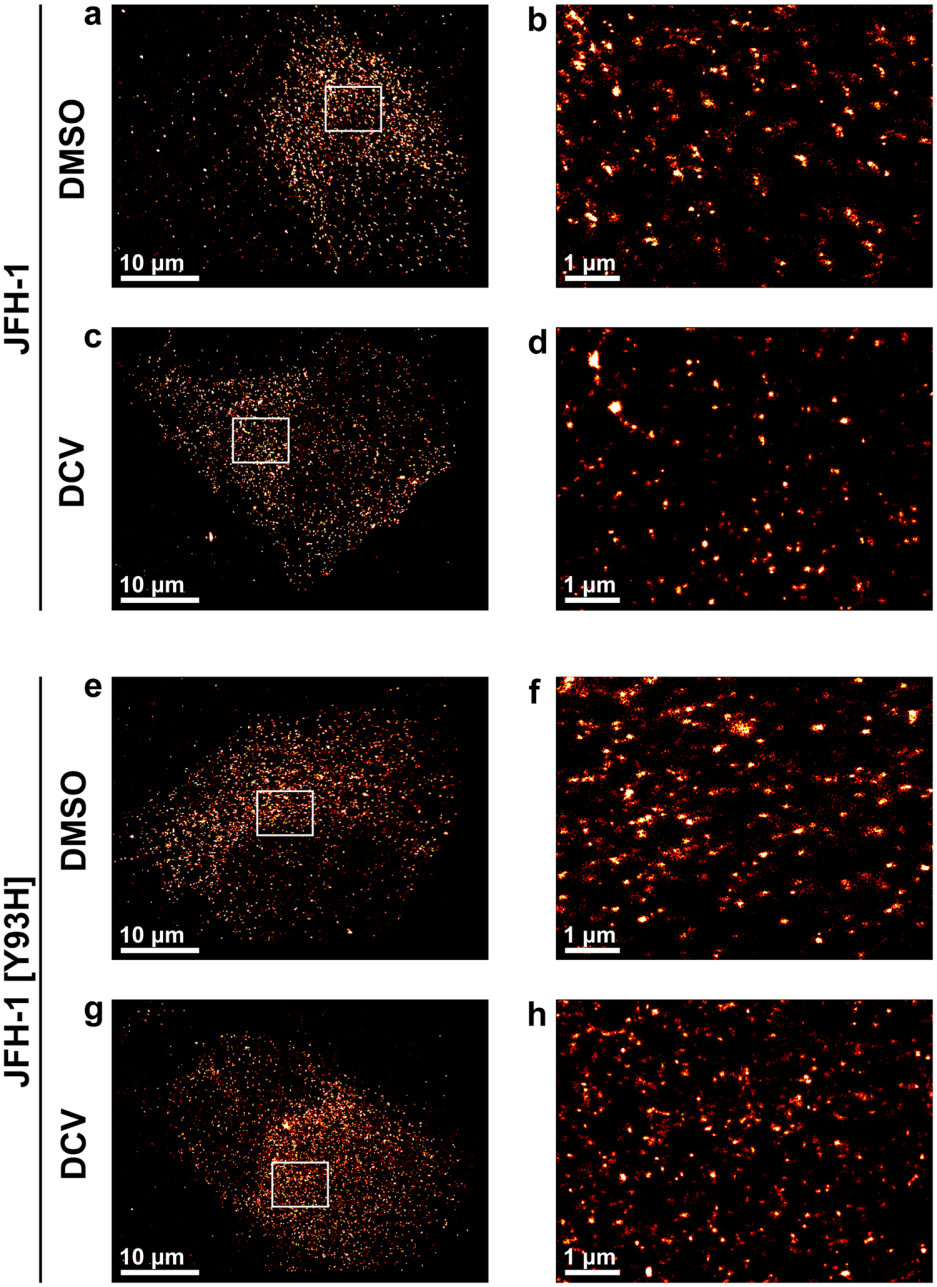
NS5A localisations remain clustered after treatment with daclatasvir. 3D-dSTORM images of Huh7 cells 24 hours post-infection with either JFH-1 or JFH-1 [Y93H], treated for 8 h with either DMSO or 1 nM DCV. (**a,c,e,g**) Projections of 2 μm-thick volumes, 100 nm histogram bins. (**b,d,f,h**) Regions of interest within (**a**,**c**,**e**,**g**). Projections of 2 µm-thick volumes, 10 nm histogram bins. Images are sum projections over depth (*z*) smoothened with a Gaussian filter σ = 20 nm.

Having demonstrated the use of 3D-dSTORM to precisely measure the sizes of NS5A clusters, we next investigated the effect of the antiviral drug DCV, which inhibits NS5A (Fig. 5a–d). We additionally tested a DCV-resistant virus JFH-1 [Y93H]^48,49^ (Fig. 5e–h), expecting to observe a different response to the drug. This mutant is reported to exhibit > 1,000-fold increase in resistance, although this resistance is linked to a loss of progeny virion production^48^. NS5A localisations after infection with the Y93H mutant virus were organised into clusters with similar shape and size range as with JFH-1, indicating that the JFH-1 [Y93H] virus strain retained the ability to form active replication complexes.

**Figure 6:**
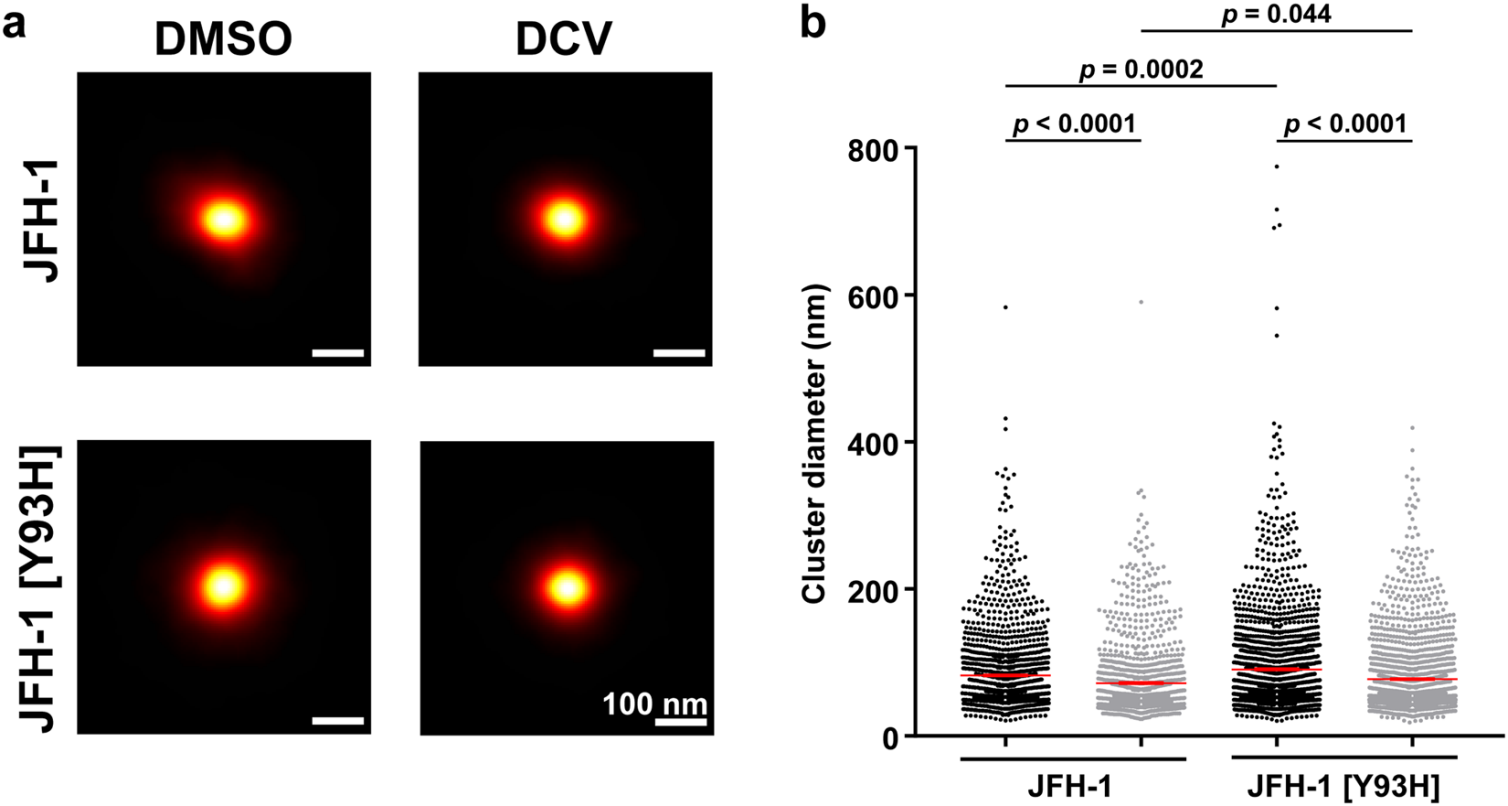
DCV treatment of JFH-1 or JFH-1 [Y93H] infected cells reduces NS5A cluster size. (**a**) Average clusters of NS5A from JFH-1 or JFH-1 [Y93H] infected cells treated with either DMSO or DCV. Sum projections over depth (*z*), 4 nm histogram bins. (**b**) NS5A cluster diameters, showing mean and standard error of the mean. Statistical significance determined by ordinary one-way ANOVA.

Treatment with DCV did not affect the average organisation of NS5A into clusters (Fig. 6a) in cells infected with JFH-1, but resulted in a significant reduction in their diameter (*F* = 31.53, *p* < 0.0001, ordinary one-way ANOVA) (Fig. 6b). Mean cluster diameter decreased by 10 nm, from 82.5 ± 1.3 nm for the control (mean ± SEM; *n* = 1538) to 72.0 ± 1.4 nm (*n* = 1266) after treatment with DCV. Despite its resistance to DCV, the mean diameter of Y93H mutant NS5A clusters also reduced significantly following DCV treatment by 13 nm, from 90.5 ± 1.5 nm (*n* = 1904) to 77.4 ± 1.2 nm (*n* = 1717) (Fig. 6b). Although NS5A clusters from both virus strains were altered by DCV, the resistant virus produced clusters larger than JFH-1 in the absence of DCV treatment (Fig. 6b), an observation that has been reported by others^26^. These findings suggest that DCV alters the organisation of NS5A within clusters to a distribution more closely resembling NS3 in both wild type and resistant virus strains.

**Figure 7:**
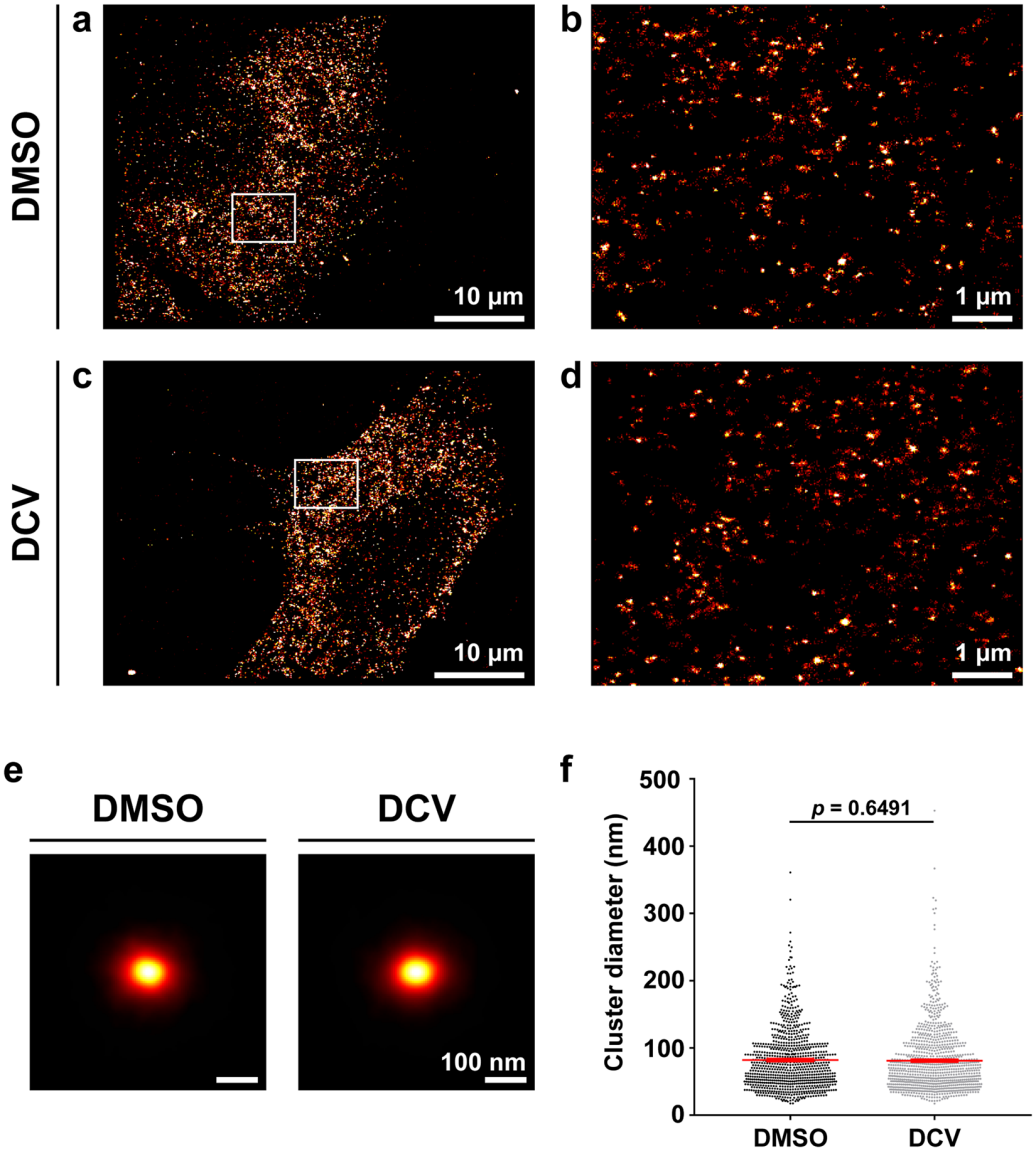
DCV treatment of JFH-1 infected cells does not change NS3 cluster sizes. 3D-dSTORM images of Huh7 cells 24 hours post-infection with JFH-1, treated for 8 h with either DMSO or DCV. (**a**,**c**) Projections of 2 µm-thick volumes, 100 nm histogram bins. (**b**,**d**) Regions of interest within (**a**,**c**). Images are sum projections over depth (*z*) smoothened with a Gaussian filter σ = 20 nm. (**e**) NS3 cluster diameters. Mean and standard error of the mean are shown. Statistical significance (not significant) determined by two-tailed Student’s *t*-test with Welch’s correction.

To confirm that this phenotype was restricted to the organisation of NS5A, the size of NS3 protein clusters by 3D-dSTORM was investigated following DCV treatment (Fig. 7). NS3 protein clusters were observed throughout the cytoplasm of infected cells (Fig. 7a–d) comparable to observations with NS5A. After DCV treatment there was no significant difference (*t* = 0.46, *p* = 0.65, Student’s *t*-test with Welch’s correction) in cluster diameter (81.0 ± 1.6 nm; mean ± SEM; *n* = 979) compared to the NS3 clusters treated with DMSO control (82.0 ± 1.6 nm; *n* = 854).

## Discussion

The data presented here provide insight into the organisation of HCV NS proteins at the ultrastructural level using SMLM. We have discovered that there are significant differences between the organisation of NS5A and NS3 molecules into clusters, with NS3 forming smaller clusters. Surprisingly, DCV had a similar effect on both wild type and resistant mutant viral strains, reducing the sizes of clusters in both cases.

The DBSCAN algorithm used in our analysis to segment and analyse clusters of localisations identified clusters by detecting density differences between clustered points and the background^43^. This approach has been widely used in other SMLM studies, such as the analysis of syntaxin clusters^50^, the distribution of RNA polymerase within the nucleus^51^, the organisation of C-type lectins^52^ and the protein kinase RAF in cancer signaling^53^. The DBSCAN algorithm and others such as Voronoi tessellation^54^ and model-based Bayesian approaches^55^ are more useful for analysing the types of clusters obtained here, as they provide a methodology to measure the sizes of individual clusters, and measurements are less dependent on cluster shape and size. While other clustering approaches have been used to analyse SMLM data such as Ripley’s functions^56^ or pair correlation^44^, these methods provide an overall measure of clustering for the region of interest and do not provide information on a per-cluster basis.

Our SMLM data revealed numerous clusters of NS5A and NS3 localisations within the diffraction-limited foci observed by wide-field. The cluster sizes fit well within the reported size of DMVs produced during HCV infection as observed by EM^12,17^. We expect that these clusters represent NS proteins associated with DMVs within infected cells^12^. Live-cell imaging studies have previously identified two-populations of NS5A, small and large (>1.0 µm) structures, which correspond to fast and slow moving structures, respectively^57,58^. Observations in this study indicate that the larger fluorescent structures observed in wide-field result from the overlapping fluorescent signals of several discrete structures in close proximity. These findings agree with another investigation using super-resolution microscopy which also did not detect these two distinct species^59^.

A surprising observation by 3D-dSTORM was the discrepancy in size between NS3 and NS5A protein clusters. This was unexpected as both proteins are known to be involved with, and organised into the replication complex^5^ and confocal fluorescence microscopy has shown that they colocalise^12,16^. One interpretation of the data is that each NS protein is associated with the replication complex in a unique way, perhaps through differential organisation of the NS proteins within the DMVs. Alternatively, each NS protein may associate with mutually exclusive DMV structures that exist in close proximity, in structures which were previously unresolvable by conventional microscopy approaches.

Our observations with SGR harbouring cells revealed clusters of NS5A larger than our results from HCV infected cells. SGR harbouring cell lines stably produce HCV replication complexes and likely contain long-lived structures which have been shown previously to develop into large multi-vesicular structures^12^. NS5A is localised to lipid droplets by an interaction with core, therefore this phenotype is not observed in SGR harbouring cells^21^. We conclude that the size difference between NS5A and NS3 clusters observed in HCV infection are not due to the localisation of NS5A to lipid droplets.

We demonstrated that short treatments (8 h) with DCV reduced the size of NS5A clusters. The treatment was applied before DCV is reported to inhibit HCV replication but after it disrupts virion assembly^28^. Therefore, this reduction in cluster phenotype likely corresponds to an inhibition of an NS5A function separate from RNA replication, such as an interaction between NS5A and core, the virion capsid protein^21,60^. This is supported by a recent study which found that DCV disrupted the delivery of HCV RNA to virus assembly sites^26^. Interestingly, a significant difference in the average size of NS5A clusters was observed in the Y93H resistant variant under DMSO control conditions compared to wildtype. This phenotype has been reported previously^26^ and may result from the fitness cost associated with harbouring the resistance mutation^49^.

The reduction in NS5A cluster size was conserved in the presence of the Y93H resistance associated substitution despite >1,000 increase in EC_50_ against DCV with JFH-1^48,49^. We additionally confirmed this phenotype is specifically associated with NS5A after no significant difference was observed for NS3 clusters following DCV treatment. Although DCV disrupts HCV RNA delivery to the virus assembly site in wild type, the Y93H mutant strain retains colocalisation of HCV RNA with core and NS4B during DCV treatment^26^. Therefore, the reduction in NS5A cluster size observed in this study is not solely responsible for the inhibition of HCV replication observed with DCV.

In conclusion, the results presented here further our insight into the organisation of HCV NS proteins within infected cells, with observations previously inaccessible by conventional microscopy approaches. The field of SMLM is continually developing and a multi-colour approach will improve the current model of the NS protein organisation within replication complexes.

## Methods

### Cell culture

Huh7 cells^61^ were cultured in Dulbecco’s modified Eagle’s medium (Sigma) supplemented with 10% (v/v) foetal bovine serum, 100 IU/ml penicillin, 100 µg/ml streptomycin, and 1% (v/v) non-essential amino acids (Lonza) in a humidified incubator at 37 °C with 5% CO_2_.

### Infectious virus propagation

*In vitro* transcribed full-length JFH-1 or JFH-1 [Y93H] virus RNA was produced using the T7 RiboMAX Large Scale RNA Production System (Promega) from 1 µg of *XbaI* digested and mungbean nuclease (NEB) treated pJFH-1 and pJFH-1 [Y93H] DNA constructs. *In vitro* transcribed RNA was purified by phenol/chloroform extraction and stored at −80 °C. Cell culture derived virus was produced from electroporation of 8 × 10^6^ Huh7 cells in ice-cold diethyl pyrocarbonate (DEPC)-phosphate-buffered saline (PBS) with 10 µg of *in vitro* transcripts using a square-wave protocol at 260 V for 25 ms. Cells were immediately recovered into media and 2 × 10^6^ cells seeded into a T175 culture flask. Cell culture supernatant was collected and replaced with fresh media every 24 h and virus stocks stored at −80 °C.

### Virus titration

Virus titre was determined as described previously^62^. In brief, supernatants were titrated in a 2-fold dilution series onto Huh7 cells seeded at 8 × 10^3^ cells/well 8 h prior in a 96 well plate. The cells were incubated under normal cell-culture conditions for 48 h before washing in PBS and fixation in 4% paraformaldehyde (PFA) for 20 min. Cells were permeabilised in 0.2% Triton X-100 in PBS for 10 min and stained with anti-NS5A serum^63^ (1:2,000) and Alexa Fluor 594 donkey anti-sheep secondary antibody for 2 h at room temperature. Infected cells were counted using the IncuCyte ZOOM platform and virus titres determined from the average of 3 or more adjacent wells.

### Fluorophore conjugation of primary antibodies

Monoclonal primary antibodies, NS5A (9E10; gift from Timothy Tellinghuisen), NS3 and NS5A (Virostat; 1877), were labelled with Alexa Fluor 647 NHS ester (Life Technologies) at an antibody/dye ratio of ~1:1 (see Supplementary Table 1). Fluorescent dye (0.1 µg) was incubated with 3 µl primary antibody (1 mg/ml) and 125 mM NaHCO_3_ in PBS for 30 min in the dark. Labelled antibodies were recovered from excess unreacted fluorescent dye using 40 K MWCO Zeba Spin Desalting columns (Thermo Fisher Scientific) following the manufacturer’s instructions. Fluorescence labelling of antibodies was confirmed by measuring the absorbance trace at 280 nm and 665 nm.

### dSTORM sample preparation

1 × 10^5^ Huh7 cells were seeded onto 25 mm diameter round glass coverslips (Warner Instruments) pre-cleaned in a 1:1:5 solution of ammonium hydroxide: hydrogen peroxide: ddH_2_O at 80 °C for 16 h. Cells were infected at an multiplicity of infection of 0.1 for 24 h before fixation with 4% PFA in PBS and permeabilisation in 0.2% Triton X-100. Samples were blocked in 1% normal donkey serum in PBS for 1 h, then stained with fluorophore conjugated monoclonal primary antibodies against either NS5A (0.2 µg/ml) or NS3 (0.4 µg/ml). Labelled antibodies were used at 1:2000 in PBS, following an assessment of labelling conditions The Virostat NS5A antibody was used at 1:1000 in PBS. (see Supplementary Fig. S2).

### dSTORM microscopy system

Super-resolution imaging was performed on a custom-built system based on the 3D-PALM apparatus of York *et al*.^34^, described previously^64^. Our system used an inverted microscope (IX81, Olympus) with a water-immersion 60 × 1.2 NA objective lens (UPLSAPO60XW, Olympus). Wide-field laser illumination was provided at 642 nm and 405 nm wavelengths (Light Hub, Omicron Laserage). Sample drift in *z* during image acquisition was mitigated by a focus locking device (C-focus, Mad City Labs). Image magnifiers of 1.6 × , 1.2 × , and a cylindrical lens (focal length 150 mm) were used prior to capture on an electron-multiplying CCD camera cooled to −80 °C (iXon Ultra, Andor), using previously published scripts^34^.

### dSTORM image acquisition and processing

Before image capture, samples were incubated with 0.01% poly-L-lysine (Sigma) for 10 min and then incubated with 150 nm gold nanoparticles (Sigma) diluted 1:25 in PBS for use as fiducial markers as described previously^64^. Calibration images of the point spread function (PSF) over a 4 µm range in 50 nm steps were taken from selected fiducial markers. Data was collected in the presence of oxygen scavenging buffer consisting of glucose oxidase and catalase (10 and 50 U, respectively; Sigma), 12.5 mg/ml D-glucose, and 1 mM 2-mercaptoethylamine (Sigma) in PBS (pH 8.0). Oxygen scavenging buffer was replenished every hour with a freshly prepared solution. Fluorophores were stochastically activated under wide-field illumination with 642 nm and 405 nm lasers at 50 mW and up to 1.6 µW, respectively. Multiple data sets of 11,000 raw images were acquired at a frame rate of 20 Hz and camera gain of 100. Fluorescent events were localised in *x-y* and *z* by cross-correlation with the PSFs captured in the calibration file, and corrected for drift by tracking the gold nanoparticles using *palm3d* software^34^ (see https://github.com/AndrewGYork/palm3d). Localisations with a cross-correlation with the calibration PSF below 0.4 were rejected^34^.

### dSTORM image analysis

Localised fluorescent events were binned into histograms for display and corrected for distortion by the cylindrical lens. The *x-y-z* localisation precision was routinely measured^65^ for fiducial markers (see Supplementary Table 1). 3D Gaussian smoothing was applied in FIJI to represent the localisation precision, and *z-*stacks were displayed in FIJI using the Red Hot look-up table. Image segmentation by DBSCAN (density based spatial clustering of applications with noise) from the scikit-learn library^66^, and cluster measurements were conducted in Python using custom written scripts (see https://github.com/Christopher-Bartlett/palm3d_dbscan). Localisation coordinates from *palm3d* output files were extracted into a NumPy array and fiducial marker localisations were removed by filtering for localisations with greater than 1,000 neighbours within a 40 × 40 × 60 nm *x*-*y*-*z* box. Localisations were then clustered using the DBSCAN algorithm with a search radius (ε) of 150 nm and minimum samples of 30. Graphical and statistical analysis of cluster measurements was conducted in GraphPad Prism using either Student’s *t-*test with Welch’s correction or an Ordinary One-Way ANOVA with multiple comparisons using Tukey’s correction.

## Electronic supplementary material

Supplementary Dataset 1

## Data Availability

The datasets generated and analysed during this study are available from the corresponding author(s) on reasonable request.
