# Supplementary material for: Visualisation and analysis of hepatitis C virus non-structural proteins using super-resolution microscopy
Source: Sci Rep. 2018 Sep 11;8:13604. doi: 10.1038/s41598-018-31861-0 (PMC6134135; doi:10.1038/s41598-018-31861-0)
Supplement: Supplementary file 1 — Supplementary Dataset 1 [file 41598_2018_31861_MOESM1_ESM.pdf]

Visualisation and analysis of hepatitis C virus non-structural proteins using super-resolution microscopy

Christopher Bartlett, Alistair Curd, Michelle Peckham, Mark Harris*

School of Molecular and Cellular Biology, Faculty of Biological Sciences and Astbury Centre for Structural Molecular Biology, University of Leeds, Leeds, LS2 9JT, UK

Corresponding authors:

Mark Harris, email: m.harris@leeds.ac.uk

Michelle Peckham, email: m.peckham@leeds.ac.uk

Sample	Antibody	Ab_{fluor}	N_{locs}	s_{fid} (nm)			N_{cl}	$n_{locs/cl}$	D_{cl} (nm)
				x	*y*	*z*			
Huh7 24 hpi JFH-1	αNS5A	1.1	151,961	17.8	15.7	52.5	350	142.0 ± 8.6	105.1 ± 3.7
			217,017	10.3	10.8	36.0	398	146.4 ± 7.6	81.4 ± 2.3
			143,262	11.1	11.8	42.0	143	162.1 ± 18.0	88.4 ± 2.9
	αNS3	0.8	144,839	22.2	23.0	122.9	356	98.5 ± 4.6	56.0 ± 1.3
			90,358	10.8	9.8	54.2	218	70.4 ± 3.5	52.9 ± 1.6
			277,315	34.5	21.5	111.6	369	97.0 ± 4.5	79.9 ± 2.1
Huh7 24 hpi JFH-1 + 8 h DMSO	αNS5A	1.2	245,248	13.3	15.1	52.8	601	171.0 ± 9.7	100.0 ± 2.3
			239,968	23.9	15.7	61.8	474	190.3 ± 10.5	81.9 ± 2.6
			147,165	26.4	14.2	99.3	463	103.4 ± 4.1	60.2 ± 1.4
HuH7 24 hpi JFH-1 + 8 h DCV		1.4	244,218	7.3	16.9	34.9	562	184.7 ± 13.8	70.0 ± 2.1
			277,811	9.0	8.6	25.7	510	195.9 ± 10.7	77.5 ± 2.4
			93,305	14.3	13.5	55.9	194	190.3 ± 65.7	63.1 ± 2.5
Huh7 24 hpi JFH-1 [Y93H] + 8 h DMSO	αNS5A	1.4	246,876	12.7	9.4	53.9	529	160.3 ± 13.2	89.0 ± 2.1
			311,709	24.7	20.8	112.3	697	198.2 ± 12.9	110.5 ± 3.3
			215,817	8.8	11.8	37.3	678	132.6 ± 7.1	71.1 ± 1.8
Huh7 24 hpi JFH-1 [Y93H] + 8 h DCV		1.2	161,712	17.6	11.7	80.5	331	125.5 ± 6.2	67.5 ± 2.3
			194,281	12.9	13.6	72.1	450	135.1 ± 7.4	70.9 ± 2.1
			355,632	18.7	17.0	91.4	936	154.9 ± 9.8	84.1 ± 1.8
SGR-Neo-JFH-1 (Stable)	αNS5A	1.0	157,597	13.9	18.9	45.4	285	153.7 ± 15.9	81.4 ± 2.6
			558,773	32.3	22.8	158.4	1,066	252.7 ± 14.7	113.3 ± 2.5
			431,225	17.9	15.9	79.1	373	422.4 ± 52.6	115.5 ± 3.7
Huh7 24 hpi JFH-1 + 8 h DMSO	αNS3	0.8	148,392	31.3	25.7	193.2	150	85.4 ± 7.2	69.5 ± 2.5
			93,033	43.8	19.0	161.3	182	85.3 ± 4.6	85.1 ± 3.3
			171,157	29.1	19.3	94.8	522	100.1 ± 5.2	84.5 ± 2.2
Huh7 24 hi JFH-1 + 8 h DCV			164,060	39.2	14.4	64.0	554	94.1 ± 3.8	78.8 ± 2.1
			92,705	39.5	18.1	70.5	188	85.2 ± 7.6	96.1 ± 3.8
			101,812	30.1	19.0	134.6	237	93.2 ± 6.0	74.2 ± 2.6

Supplementary Table S1: Summary of 3D-dSTORM image characteristics. Ab_{fluor}: Ratio of fluorophore to monoclonal antibody. N_{locs}: Total number of localisations from dataset of 11,000 frames. s_{fid}: Localisation precision (standard deviation of localisations) of fiducial markers used for drift-correction. N_{cl}: Number of clusters identified by DBSCAN analysis from regions of interest (ROI). $n_{locs/cl}$: Number of localisations per cluster (mean ± SEM) in ROI. D_{cl}: Cluster diameter (mean ± SEM) in ROI.

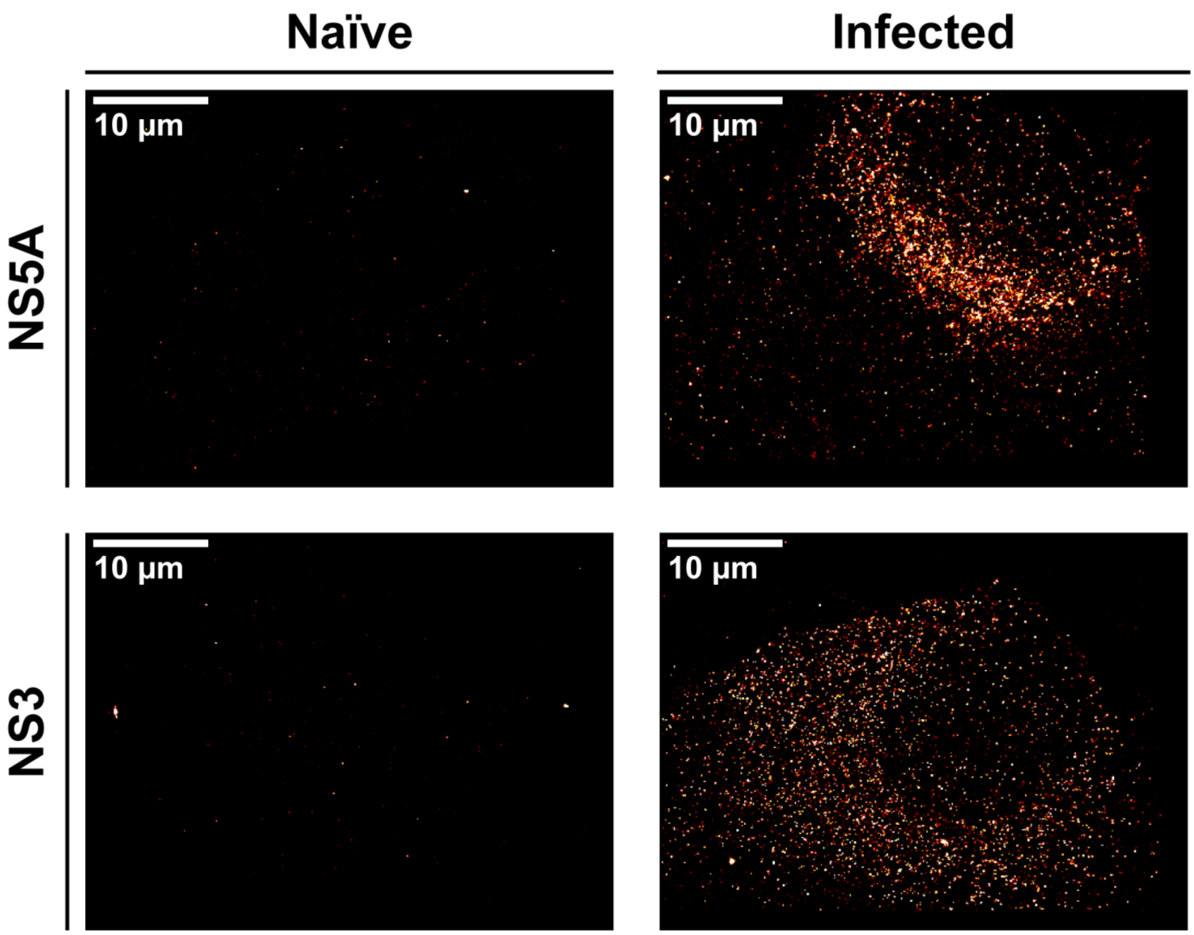

Supplementary Figure S1: Validation of antibody staining for 3D-dSTORM. 3D-dSTORM images of naïve or JFH-1 infected Huh7 cells immunostained for NS5A or NS3. Projection of 2 µm-thick volume, 100 nm histogram bins. 3D-dSTORM images are sum projections over depth (*z*) smoothened with a Gaussian filter $\sigma = 20$ nm. Histograms for naïve and infected cells are shown with the same look-up table for NS5A and NS3, respectively.

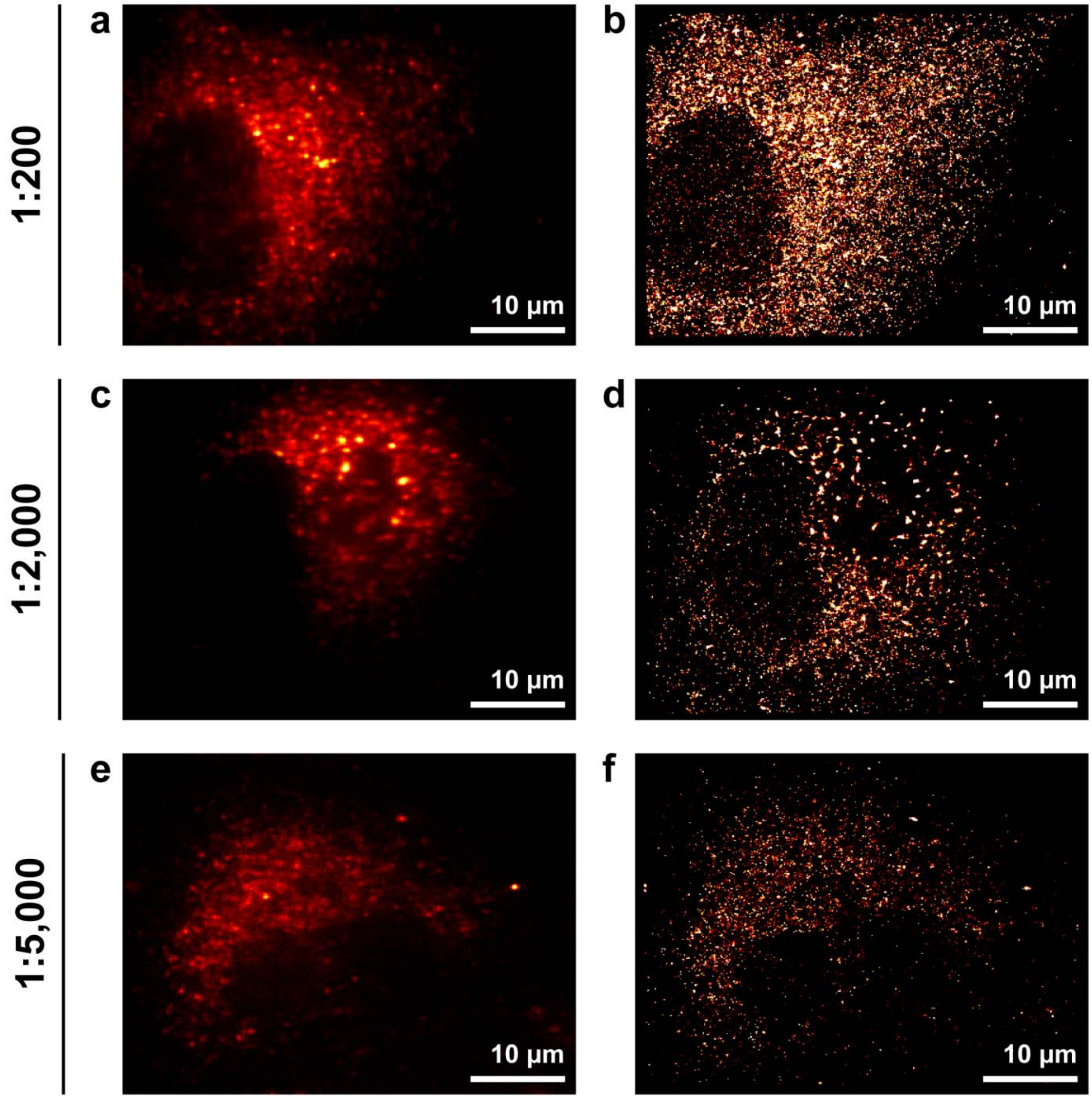

Supplementary Figure S2: 3D-dSTORM imaging of Huh7 cells infected with JFH-1 for 24 hours and immunostained for NS5A at different concentrations. (**a**, **b**) 1:200, (**c**, **d**) 1:2,000, (**e**, **f**) 1:5,000. (**a**, **c**, **e**) Wide-field fluorescence image of Huh7 cell stably harbouring SGR-Neo-JFH-1 and immunostained for NS5A at different concentrations. **(b**, **d**, **f**) 3D-dSTORM image of cell in **a**, **c**, **e**. Projection of 2 µm-thick volume, 100 nm histogram bins. 3D-dSTORM images are sum projections over depth (*z*) smoothened with a Gaussian filter σ = 20 nm.

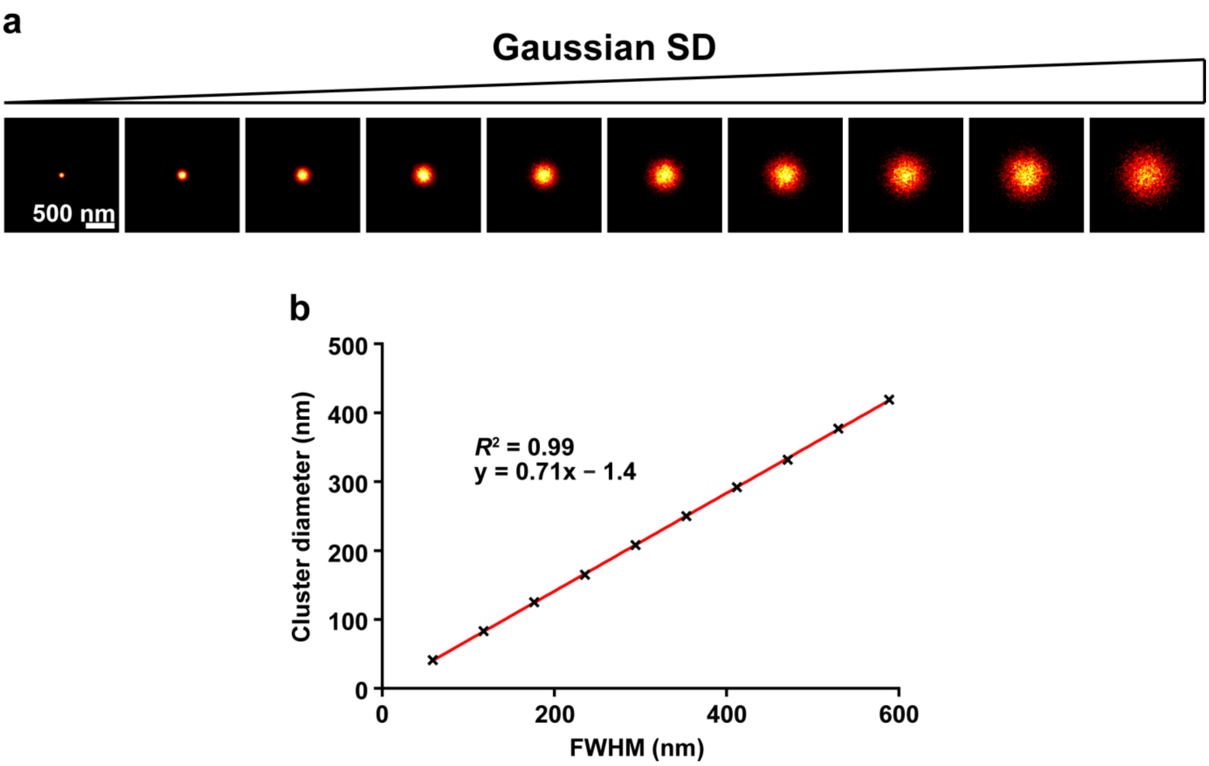

Supplementary Figure S3: Correlation between cluster diameter and FWHM. (**a**) Simulated clusters of localisations with spherical Gaussian density distributions. Gaussian standard deviation ranging from 25–250 nm, in 25 nm intervals. Images are sum projections onto the plane shown, with 4 nm histogram bins, smoothened with a Gaussian filter σ = 20 nm. (**b**) Correlation between measured cluster diameter and FWHM of simulated clusters. Goodness of fit (R^2) and the slope of linear regression are shown.

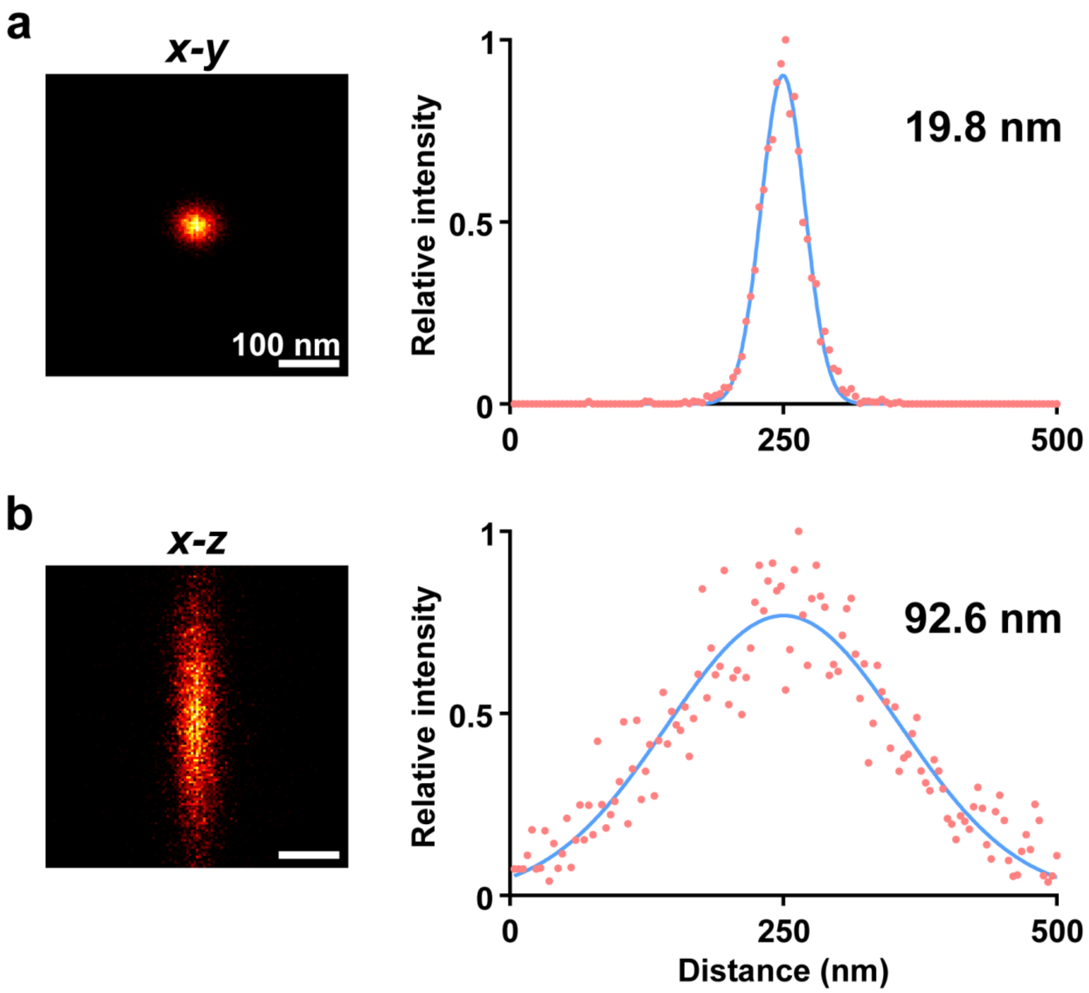

Supplementary Figure S4: Localisation precision measurement from smallest NS3 clusters. (**a**) *x-y* (**b**) *x-z*. Results of summing the smallest NS3 clusters, containing a total of 9,901 localisations (124 clusters, up to cluster diameter 45 nm, registered by their centroid). Images are sum projections onto the plane shown, with 4 nm histogram bins. Line profiles through their centre, normalised to the peak intensity and fitted with a Gaussian model, standard deviation of fit is indicated. Precision was expected to be lower in *z*, and may include contributions from intra-cellular inhomogeneity (variations in refractive index, including at the boundaries of DMVs).

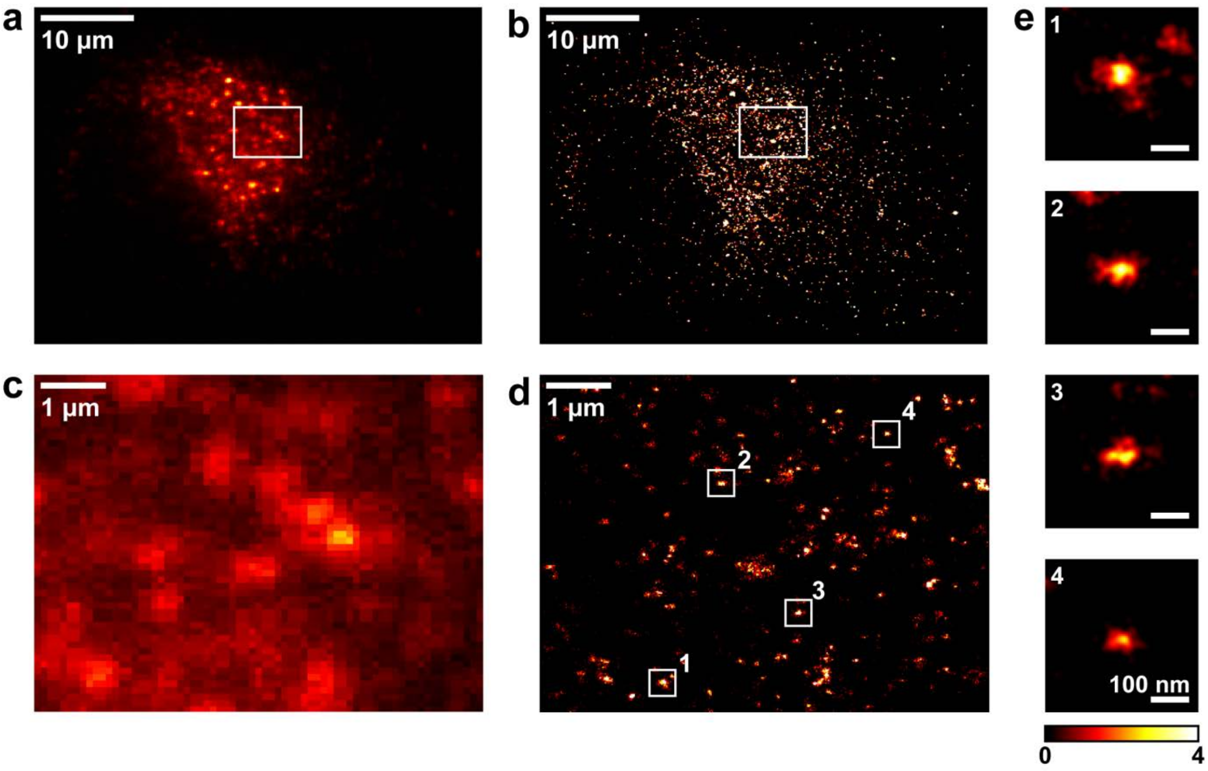

Supplementary Figure S5: 3D-dSTORM imaging of Huh7 cells stably harbouring SGR-Neo-JFH-1 and immunostained for NS5A using Virostat antibody. (**a**) Wide-field fluorescence image of Huh7 cell stably harbouring SGR-Neo-JFH-1 and immunostained for NS5A using Virostat antibody. (**b**) 3D-dSTORM image of cell in **a**. Projection of 2 µm-thick volume, 100 nm histogram bins. (**c**) Region of interest within **a** at higher magnification. (**d**) 3D-dSTORM image of region shown in **c**. Projection of 2 µm-thick volume, 20 nm histogram bins. (**e**) Individual clusters of localisations (numbered in **d**). Projections of 1 µm-thick volumes, 5 nm histogram bins. Colour bar indicates number of localisations per histogram bin. 3D-dSTORM images are sum projections over depth (*z*) smoothened with a Gaussian filter σ = 20 nm.

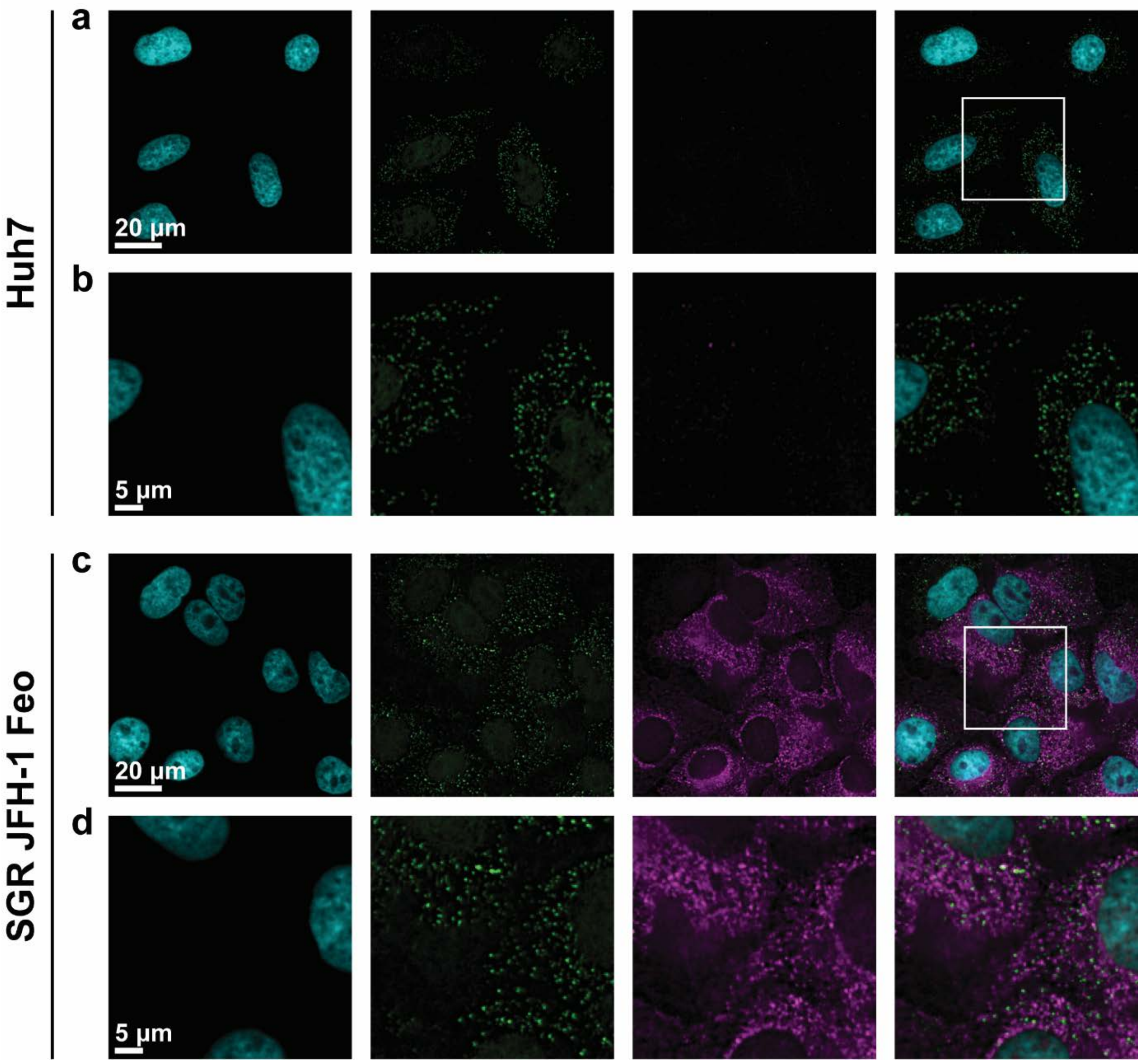

Supplementary Figure S6: Immunofluorescence imaging of naïve or SGR-Neo-JFH-1 harbouring Huh7 cell lines immunostained for NS5A and dsRNA. (**a**) Naïve Huh7 cells. (**b**) Magnified view of region of interest from white box in **a**. (**c**) SGR-Feo-JFH-1 harbouring Huh7 cells. (**d**) Magnified view of region of interest from white box in **c**. Stained with DAPI (cyan), and for dsRNA (green) and NS5A (magenta).
